# Risk Factors of Coronary Artery Disease in Secondary Prevention—Results from the Athero*Gene*—Study

**DOI:** 10.1371/journal.pone.0131434

**Published:** 2015-07-08

**Authors:** Elvin Zengin, Christoph Bickel, Renate B. Schnabel, Tanja Zeller, Karl-J. Lackner, Hans-J. Rupprecht, Stefan Blankenberg, Dirk Westermann, Christoph Sinning for the AtheroGene–Study Investigators

**Affiliations:** 1 Department of General and Interventional Cardiology, University Heart Center Hamburg, Hamburg, Germany; 2 Department of Medicine, Federal Armed Forces Central Hospital Koblenz, Koblenz, Germany; 3 Institute of Clinical Chemistry and Laboratory Medicine, University Medical Centre of the Johannes Gutenberg University Mainz, Mainz, Germany; 4 Department of Medicine II, GPR Klinikum Ruesselsheim, Ruesselsheim, Germany; Medstar Washington Hospital Center, UNITED STATES

## Abstract

**Background:**

Risk factors are important in cardiovascular (CV) medicine for risk stratification of patients. We aimed to compare the traditional risk factors to clinical variables for the prediction of secondary cardiovascular events.

**Methods and Results:**

For this study, 3229 patients with known coronary artery disease (CAD) were included. We calculated whether the traditional risk factors, diabetes mellitus, increased LDL/HDL ratio, arterial hypertension and smoking alone and in combination with the clinical variables, ejection fraction, creatinine clearance, multi-vessel disease and CRP concentration predict the outcome cardiovascular death or non-fatal myocardial infarction (N = 432) during the mean follow-up time of 4.2 ± 2.0 years. In this cohort diabetes mellitus was the risk factor with the strongest influence regarding occurrence of secondary events (hazard ratio; HR:1.70, confidence interval; CI 95%: 1.36-2.11; P<0.0001), followed by LDL/HDL ratio and smoking. However, risk stratification is further improved by using additional clinical variables like ejection fraction (HR:3.30 CI 95%:2.51-4.33; P>0.0001) or calculated creatinine clearence (Cockroft-Gault formula) (HR:2.26 CI 95%:1.78-2.89; P<0.0001). Further ameliorating risk stratification from the clinical variables were CRP and multi-vessel disease. The most precise risk prediction was achieved when all clinical variables were added to the CV risk factors.

**Conclusion:**

Diabetes mellitus has the strongest influence to predict secondary cardiovascular events in patients with known CAD. Risk stratification can further be improved by adding CV risk factors and clinical variables together. Control of risk factors is of paramount importance in patients with known CAD, while clinical variables can further enhance prediction of events.

## Introduction

Secondary prevention in coronary artery disease (CAD) is a major task to improve the prognosis of patients with cardiovascular disease. Moreover, risk factor control is still not achieved in many patients with CAD as current results of EUROASPIRE IV show [1]. Nevertheless, effective prevention strategies would result in reduced recurrent events [2–9] and data of previous prevention studies show that nine easily accessible risk factors account for about 90% of the risk of acute myocardial infarction [10]. In recent years, studies from all over Europe, especially EUROASPIRE I,II and III [11–13], could demonstrate that patients after a coronary event lack the proper life style changes and more importantly lack appropriate medical treatment of modifiable risk factors, like diabetes mellitus [11–16]. Indeed there is evidence that a cohort with an especially adverse outcome in secondary prevention is patients with diabetes mellitus [5, 17, 18]. In these patients, lower targets for LDL cholesterol with 70 mg/dL and moderate blood pressure with a systolic value below 140mmHg are suggested to reduce the additional risk in comparison to CAD patients without diabetes mellitus [19–24]. The prediction of models based on traditional CV risk factors can be augmented by the use of clinical variables known from the index event. In this context, ejection fraction of the left ventricle, the number of diseased coronary vessels, the concentration of C-reactive protein and the renal function can, when combined with traditional CV risk factors, enhance risk prediction [25–29].

The aim of study was to demonstrate the influence of combining CV risk factors and clinical variables to improve risk prediction in terms of secondary prevention in patients with known CAD.

## Methods

### Study cohort—Athero*Gene*

Between June 1996 and March 2004, 3229 patients who presented with chest pain at the second department of Medicine of the University Medical Centre of the Johannes Gutenberg University Mainz or the Federal Armed Forces Central Hospital Koblenz were enrolled in the Athero*Gene* Study. The AtheroGene study is a prospective, multicenter, clinical cohort study including patients with known CAD (at least one stenosis over 30%) as diagnosed by coronary angiography. The cohort covers the whole spectrum of patients with acute coronary syndrome (ACS) and stable angina (SAP). The aim of the Athero*Gene* study was to elucidate clinical, genetic and laboratory factors associated with progression of coronary atherosclerosis and outcome during follow-up.

The mean follow-up time was 4.2 ± 2.0 years. Medical technicians did follow-up the patients by questionnaires. The outcome parameters death from cardiovascular causes or non-fatal myocardial infarction during the follow-up period was reported in 432 patients. Death from causes not related to heart disease was recorded in 106 patients. Information on the cause of death was obtained from hospital or general practitioner charts.

Exclusion criteria for patients were evidence of hemodynamic shock, significant valvular heart disease, surgery or trauma within the previous month, known cardiomyopathy, known malignancies, febrile conditions, known chronic inflammatory diseases, known renal failure (increased creatinine >2.1 mg/dL) or use of oral anticoagulant therapy within the previous four weeks.

Patients with stable angina (N = 2652) were compared to the overall study cohort (N = 3229) including ACS patients. The cohort of patients with stable angina was analysed separately to investigate the influence of risk factors and clinical variables on outcome in a cohort without acute coronary syndrome (ACS). The intention of this approach was to investigate if patients with stable disease need the same risk factor control as those subjects suffering an acute cardiovascular event in the medical history. The study was approved by the review board of the Johannes Gutenberg-University Mainz. Participation was voluntary, and each subject gave written, informed consent.

### Traditional cardiovascular risk factors

Arterial Hypertension was defined as mean blood pressure of 140mmHg (systolic) over 90mmHg (diastolic). Subjects taking medication because of arterial hypertension were also classified to have arterial hypertension, even when blood pressure was controlled. Smoking was defined as ever smoking or never smoking (cessation before 40 years or no smoking at all). Diabetes mellitus was defined in regard to oral blood glucose lowering therapy or substitution of insulin. Hyperlipoproteinemia refers to patients with diagnosis of hyperlipoproteinemia by a general practitioner or LDL/HDL ratio above 3.5. Positive family history of myocardial infarction is a first degree relative with myocardial infarction below 60 years (men) or below 65 years (women).

### Clinical variables

Ejection Fraction was measured during cardiac angiography in 2294 patients by ventriculography and estimation of systolic and diastolic chamber volume. The creatinine clearence was calculated according to the formula by Cockroft-Gault [30]. The concentration of C-reactive protein was determined by a highly sensitive, latex particle-enhanced immunoassay (Roche Diagnostics; range of detection 0.1–20 mg/L; interassay coefficient of variation, 1.0% for values of 15 mg/L and 6.5% for values below 4 mg/L). As a cut-off to define patients with a high risk profile we used 3 mg/L [31]. Although this cut-off was suggested for intermediate to high risk subjects in the general population, our aim was to show that CRP might improve risk stratification by application in CAD patients. Multivessel disease was defined as two or three vessels with coronary artery disease in contrast to one affected vessel.

### Statistical analyses

Continuous variables are presented as median and 25^th^ and 75^th^ percentile, and discrete variables are presented as absolute and relative frequencies per category. Analyses were performed according to stratification by the study endpoint. For statistical testing, in the case of skewed variables, the Mann-Whitney-U-Test was used and in case of variables with a normal distribution the T-Test was applied.

For analysis, the risk factors were divided into dichotomous variables and the patients in those with and without the risk factor. Cox proportional regression analysis was used to adjust for baseline risk factors (age and sex) representing the baseline model. Second, the model was adjusted for the traditional risk factors when testing each of the risk factors in an own model (excluding the tested risk factor) to represent the cardiovascular risk factor model. The clinical variables were tested in a model adjusting for sex, age and all traditional risk factors. Third, the C-index was calculated and the effect of each risk factor in addition to the baseline model and cardiovascular risk factor model listed in tables. As for the hazard regression analysis, the clinical variables were tested in a model including age, sex and all traditional risk factors.

For further assessment of the models evaluated, net reclassification improvement (NRI) and integrated discrimination improvement (IDI) were calculated. The NRI focuses on reclassification tables and the corresponding movement of subjects according to their status regarding events. For reclassification we chose the 10 year risk categories of CVD [22]. If a model proposes a better risk stratification, subjects should move to a higher risk class if an event is likely and on the other hand move down a risk class when no event occurred [32]. An advantage of the IDI is that it does not require categories and is not based on ranks like the C-index, rather it focuses on integrated sensitivity and 1-specifity for all possible cut-off values of the model with and without the marker [32].

Analyses were performed using R 3.12.0. R: A language and environment for statistical computing. R Foundation for Statistical Computing, Vienna, Austria. ISBN 3-900051-07-0, URL http://www.R-project.org.).

## Results

### Baseline characteristics

Baseline comparison of the patients with no event and those with cardiovascular death or non-fatal myocardial infarction during follow up is shown in **Table 1**. Patients with an event were older than patients presenting without event. Incidence of diabetes mellitus and patients with LDL/HDL ratio above 3.5 was higher in the event cohort, while hypertension and smoking was similar in both groups. In terms of medication, patients without an event had higher rates of statins and beta-blockers. Ejection fraction was lower in the event cohort, while incidence of multi vessel disease (3 vessel disease) was higher in the event group.

**Table 1:** Baseline Characteristics in the Athero*Gene* study N = 3229.

Variable	No Event (2797)	CV Event (432)	All (3229)	P-Value
Sex (male)	2154 (77.0%)	318 (73.6%)	2472 (76.6%)	0.14
Age (years)	61.6± 10.0	64.0±10.2	61.9±10.0	< 0.0001
BMI (kg/m²)	27.5±3.9	27.2±4.0	27.5±3.9	0.13
Creatinine (mg/dL)	0.98 (0.86/1.11)	1.07 (0.91/1.25)	0.99 (0.86/1.12)	< 0.0001
**Traditional Risk Factors**				
Diabetes mellitus (yes)	442 (15.8%)	113 (26.2%)	555 (17.2%)	< 0.0001
Hypertension (yes)	2092 (74.8%)	325 (75.2%)	2417 (74.9%)	0.90
LDL/HDL ratio (> 3.5)	637 (22.8%)	143 (33.1%)	780 (24.2%)	< 0.0001
Smoking	1760 (62.9%)	283 (65.5%)	2043 (63.3%)	0.33
**Medication**				
Beta-blocker treatment	1801 (64.4%)	241 (55.9%)	2042 (63.3%)	0.00083
ACE-inhibitor treatment	1403 (50.2%)	249 (57.8%)	1652 (51.2%)	0.0038
Statin treatment	1311 (46.9%)	154 (35.7%)	1465 (45.4%)	< 0.0001
**Clinical Variables**				
eGFR (Cockroft-Gault) Cockroft-Gault Equation	87.0 (69.7/107.7)	73.7 (55.8/93.4)	85.1 (68.0/106.2)	< 0.0001
Ejection Fraction (%)	63.7±14.8	56.3±18.4	62.7±15.5	< 0.0001
Number of diseased vessels				
0	30 (1.1%)	1 (0.2%)	31 (1.0%)	0.16
1	786 (28.1%)	79 (18.3%)	865 (26.8%)	< 0.0001
2	836 (29.9%)	132 (30.6%)	968 (30.0%)	0.83
3	1143 (40.9%)	220 (50.9%)	1363 (42.2%)	0.00011
CRP (mg/L)	3.41 (1.52/9.01)	5.34 (2.29/14.80)	3.60 (1.60/9.68)	< 0.0001

### Cox proportional hazard regression

Cox proportional hazard regression analyses are shown in **Table 2**for the overall cohort and in **Table 3**for the SAP cohort. Diabetes mellitus increased the risk of secondary cardiovascular events and showed a hazard ratio (HR) of 1.7 in the overall cohort (HR = 1.7 in SAP patients) both with a significant result (p<0.001). Further predictive was the LDL/HDL ratio above 3.5 with HR = 1.6 in the overall cohort (HR = 1.8 in SAP patients) (both p<0.001) and ever smoking with HR = 1.3 in the overall cohort and HR = 1.4 in SAP patients (both p<0.01).

**Table 2:** Cox Proportional Hazard Regression in the overall cohort (N = 3229). Overall Cohort (N = 3229).

Variable	N	HR	Lower CI	Upper CI	p-value
LDL/HDL ratio (LDL/HDL Ratio >3.5 vs. <3.5)	3229	1.57	1.28	1.92	< 0.0001
Smoking (Smoker vs. Never-Smoker)	3229	1.34	1.09	1.66	0.0063
Diabetes Mellitus (Treated with oral medication or Insulin vs. No Diabetes)	3229	1.70	1.36	2.11	< 0.0001
Hypertension (Treated vs. not diagnosed)	3229	0.98	0.79	1.22	0.87
eGFR (eGFR >60ml/min vs. eGFR <60ml/min)	3229	2.26	1.78	2.89	< 0.0001
Ejection Fraction (EF >40% vs. EF <40%)	2294	3.30	2.51	4.33	< 0.0001
Number of dis. Vessels (One vs. Multivessel Disease)	3227	1.61	1.26	2.05	0.00016
CRP (CRP >3 mg/L vs. CRP < 3mg/L)	3196	1.63	1.33	2.00	< 0.0001

**Table 3:** Cox proportional hazard regression in the stable angina cohort (N = 2652). Stable Angina (N = 2652).

Variable	N	HR	Lower CI	Upper CI	p-value
LDL/HDL ratio (LDL/HDL Ratio >3.5 vs. <3.5)	2652	1.79	1.43	2.25	< 0.0001
Smoking (Ever-Smoker vs. Never-Smoker)	2652	1.38	1.09	1.75	0.0083
Diabetes Mellitus (Treated with oral medication or Insulin vs. No Diabetes)	2652	1.68	1.32	2.15	< 0.0001
Hypertension (Treated or diagnosed vs. Not diagnosed)	2652	0.95	0.74	1.23	0.70
eGFR (eGFR >60ml/min vs. eGFR <60ml/min)	2652	2.59	1.98	3.38	< 0.0001
Ejection Fraction (EF >40% vs. EF <40%)	1963	3.33	2.46	4.53	< 0.0001
Number of dis. Vessels (One vs. Multivessel Disease)	2651	1.43	1.09	1.88	0.0096
CRP (CRP >3 mg/L vs. CRP < 3mg/L)	2622	1.62	1.30	2.03	< 0.0001

**Fig 1:**
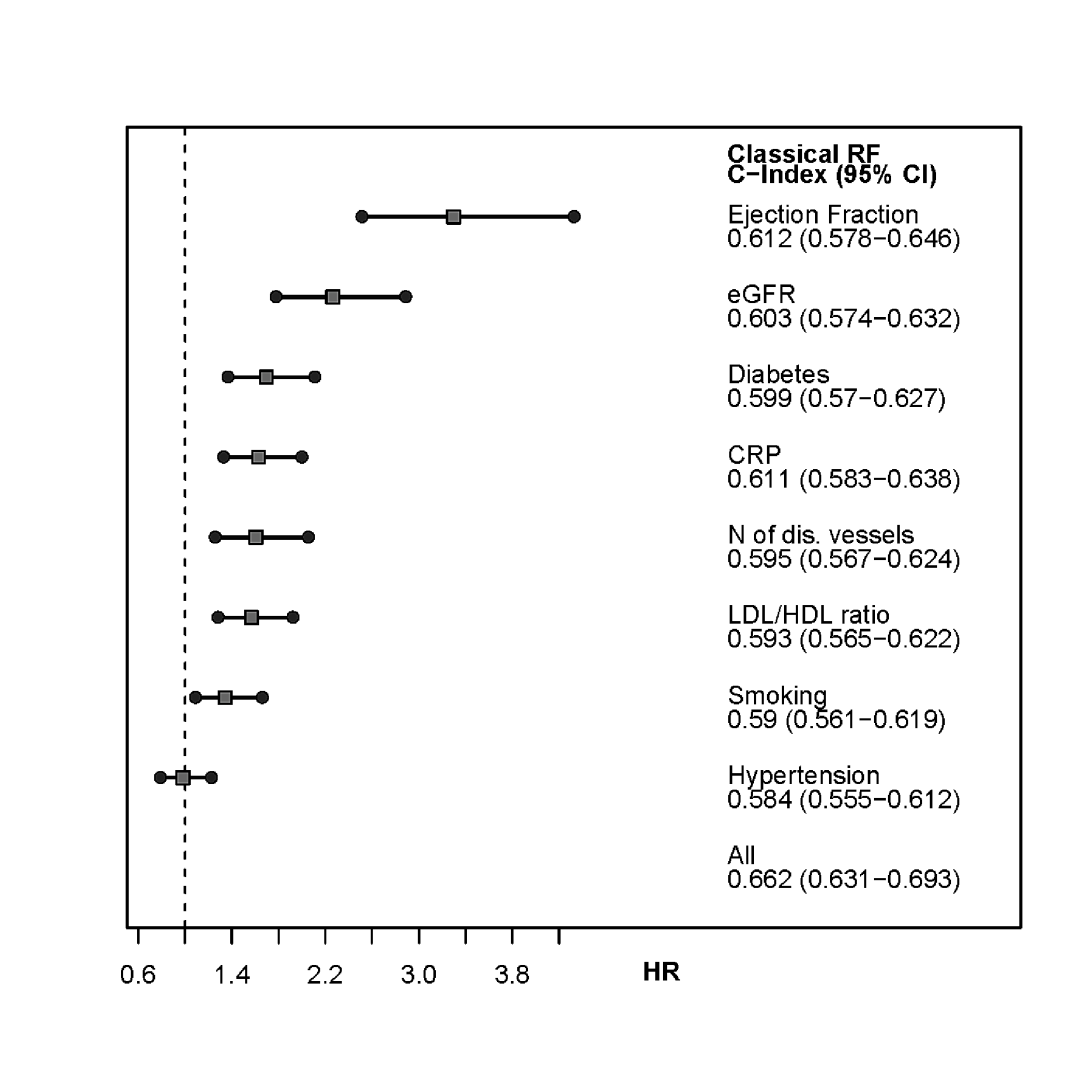
Hazard Ratio and C-Index for investigated risk factors (adjusted for age and sex) in the overall cohort. The hazard ratio and the C-Index are for the comparison cardiovascular event vs. no event during follow-up. In these figures creatinine clearance is represented by eGFR.

**Fig 2:**
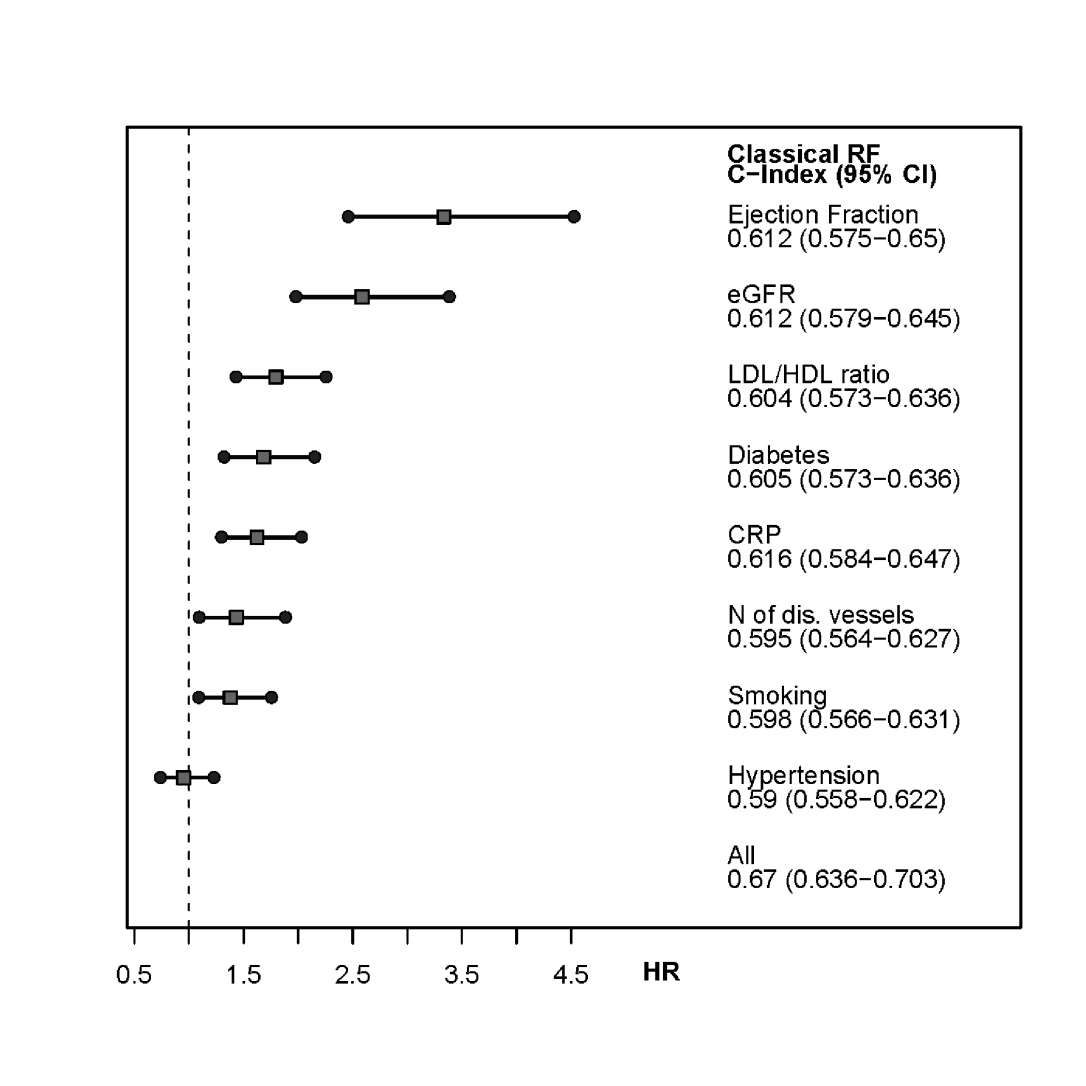
Hazard Ratio and C-Index for investigated risk factors (adjusted for age and sex) in the stable angina cohort. The hazard ratio and the C-Index are for the comparison cardiovascular event vs. no event during follow-up. In these figures creatinine clearance is represented by eGFR.

For the clinical variables evaluated in this study, the highest HR with 3.3 was shown for ejection fraction below 40% in the overall cohort (HR = 3.3 in SAP) (p for both <0.001), followed by impaired creatinine clearence with a threshold of 60mL/min with a HR = 2.3 in the overall cohort and HR = 2.6 in SAP patients (both p<0.001) and CRP with HR 1.6 (SAP HR 1.6) as well as multi vessel disease with HR 1.6 (SAP HR 1.4). The hazard ratios for the different models are presented in **Figs 1**and **2**. Arterial hypertension, a major CV risk factor, did not provide additional information regarding risk stratification in this study.

Combining all traditional risk factors with the clinical variables allowed the most precise and reliable risk stratification regarding secondary CV events (**Figs 1**and **2**).

### Calculation of the C-Index for traditional risk factors and clinical variables

Receiver operating characteristic (ROC) curve analysis was performed and the C-index calculated to identify the most important risk factors.

On top of the traditional risk factors age and sex (C-index 0.58 and 0.59 in SAP), each risk factor alone improved the C-Index with exception of arterial hypertension (**Table 4**for the overall cohort and **Table 5**for the SAP cohort). The traditional risk factor with the highest influence was diabetes mellitus with a C-index of 0.6 (0.61 in SAP), showing the best improvement of all traditional risk factors in both the overall and SAP cohort. Combining all traditional risk factors with age and sex (C-index 0.62 and 0.63 in SAP), these clinical variables increased the C-index additionally. In the overall cohort C-index for renal function, CRP and ejection fraction were all 0.64 and 0.63 for multivessel disease. In the SAP cohort, the highest C-index was shown for renal function with 0.66, followed by CRP with 0.65 and equally ejection fraction and multivessel disease with 0.64.In all, the highest C-index was achieved upon adding the traditional risk factors and the clincial variables to age and sex showing a C-index of 0.66 in the overall cohort and 0.67 in the stable angina cohort, thus allowing the most precise risk prediction.

**Table 4:** Comparison of the C-Index regarding cardiovascular events vs. no events during follow-up in the overall cohort. Overall Cohort (N = 3229).

**Variable**	**N**	**C-Index**	**Lower 95% CI**	**Upper 95% CI**	**P-Value**
Diabetes Mellitus (Treated with oral medication or Insulin vs. No Diabetes known)	3229	**0.599**	0.570	0.627	< 0.0001
LDL/HDL ratio (>3.5 vs. <3.5)	3229	**0.593**	0.565	0.622	< 0.0001
Smoking (Ever-Smoker vs. Never-Smoker)	3229	**0.590**	0.561	0.619	< 0.0001
Hypertension (Treated or Diagnosed by physican vs. Not Known)	3229	**0.584**	0.555	0.612	< 0.0001
**Variable**	**N**	**C-Index**	**Lower 95% CI**	**Upper 95% CI**	**P-Value**
eGFR (>60 mL/min vs. <60mL/min)	3229	**0.642**	0.615	0.670	< 0.0001
CRP (>3mg/L vs. <3 mg/L)	3196	**0.639**	0.612	0.666	< 0.0001
Ejection Fraction (>40% vs. <40%)	2294	**0.637**	0.604	0.670	< 0.0001
Number of dis. Vessels (Multivessel vs. 1-Vesseldisease)	3227	**0.628**	0.600	0.655	< 0.0001

**Table 5:** Comparison of the C-Index regarding cardiovascular events vs. no events during follow-up in the stable angina cohort. Stable Angina Pectoris Cohort (N = 2652).

**Variable**	**N**	**C-Index**	**Lower 95% CI**	**Upper 95% CI**	**P-Value**
Diabetes Mellitus (Treated with oral medication or Insulin vs. No Diabetes known)	2652	**0.605**	0.573	0.636	< 0.0001
LDL/HDL ratio (>3.5 vs. <3.5)	2652	**0.604**	0.573	0.636	< 0.0001
Smoking (Ever-Smoker vs. Never-Smoker)	2652	**0.598**	0.566	0.631	< 0.0001
Hypertension (Treated or Diagnosed by physican vs. Not Known)	2652	**0.590**	0.558	0.622	< 0.0001
**Variable**	**N**	**C-Index**	**Lower 95% CI**	**Upper 95% CI**	**P-Value**
eGFR (>60 mL/min vs. <60mL/min)	2652	**0.658**	0.627	0.688	< 0.0001
CRP (>3mg/L vs. <3 mg/L)	2622	**0.651**	0.621	0.681	< 0.0001
Ejection Fraction (>40% vs. <40%)	1963	**0.640**	0.604	0.676	< 0.0001
Number of dis. Vessels (Multivessel vs. 1-Vesseldisease)	2651	**0.638**	0.607	0.669	< 0.0001

### Net reclassification improvement (NRI) and integrated discrimination improvement (IDI) in the Athero*Gene* Study

The net reclassification improvement using reclassification tables based upon the proposed classification from citation [22], LDL/HDL ratio above 3.5 and ever smoking revealed an improved rate of reclassification of the overall cohort. Regarding the clinical variables, all four factors improved reclassification in risk stratification.

However, using integrated discrimination improvement, without relying on ranks like in C-index analysis, the three risk factors diabetes mellitus, LDL/HDL ratio and current smoking revealed the strongest influence on secondary event rate, affirming the results from Cox Regression analyses and the C-index analyses. According to IDI, diabetes mellitus was the strongest risk factor from the traditional risk factors with a p-value of below 0.0001, the other results are outlined in **Table 6**. The set of clinical variables, ejection fraction, creatinine clearence, CRP and multivessel disease also were strong risk predictors, improving risk stratification.

**Table 6:** Net reclassification table (NRI) and integrated discrimination improvement (IDI) in the Athero*Gene* study (N = 3229) after 5 years of follow-up.

**Traditional Risk Factors**	**IDI**	**p(IDI)**	**NRI**	**p(NRI)**
LDL/HDL ratio (>3.5 vs. <3.5)	0.0088	< 0.0001	0.055	0.021
Diabetes Mellitus (Treated with oral medication or Insulin vs. No Diabetes known)	0.0076	0.00019	0.046	0.088
Smoking (Ever-Smoker vs. Never-Smoker)	0.0028	0.0058	0.047	0.030
Hypertension (Treated or diagnosed by physician vs. Not Known)	-0.0000031	0.96	-0.0014	0.60
**Clinical Variables**	**IDI**	**p(IDI)**	**NRI**	**p(NRI)**
Ejection Fraction (40% vs. <40%)	0.035	< 0.0001	0.13	< 0.0001
Glomerular Filtration Rate (according to the Cockroft- Gault formula)	0.017	< 0.0001	0.11	< 0.0001
C-reactive Protein (>3 mg/L vs. <3 mg/L)	0.0091	< 0.0001	0.070	0.0054
Number of dis. Vessels (Multivessel vs. 1-vessel disease)	0.0055	<0.0001	0.056	0.0051

## Discussion

The results of this study from Athero*Gene* underline the importance of traditional CV risk factors for risk stratification regarding secondary prevention in a cohort of patients with known coronary artery disease. In this cohort, diabetes mellitus was the strongest traditional risk factor for prediction of future cardiovascular events. Further, patients with known CAD ever smoking or with an increased LDL/HDL-ratio had an elevated risk for cardiovascular death or non-fatal myocardial infarction. This was further enhanced when clinical variables were taken into account. Patients with stable angina and known CAD did show the same influence of traditional risk factors and clinical variables as the overall cohort regarding secondary.

### Traditional risk factors in secondary prevention

Modifiable risk factors are important in the prevention setting; other studies could already show the high influence of these factors [10–14]. Nevertheless, there is need to further improve risk prediciton [1]. Overall the results of Cox proportional regression analysis and the integrated discrimination improvement IDI analysis showed that diabetes mellitus was the risk factor with the most impact on predicting outcome. This information was further improved after combination with ever smoking and a LDL/HDL ratio >3.5. The baseline model of age and sex was augmented by the traditional risk factors to a C-index of 0.62 in the overall cohort, improving risk stratification in the Athero*Gene* Study. In the secondary prevention setting, some of the traditional risk factors like arterial hypertension and positive family history have not the same influence as reported from the primary prevention setting [3, 33–35]. From our results, arterial hypertension had no relevant influence on the secondary event rate, a fact also reported from other studies investigating the prevention of secondary events [16, 36–38]. The relevance of this has to be tested in further studies.

### Clinical variables in secondary prevention

A feasible strategy to further improve risk prediction beyond the scope of traditional risk factors is to include clinical variables into calculating future events. Therefore we included easy accessible clinical indicators known from index events in CAD patients like ejection fraction of the LV, renal function, extend of CAD at coronary angiography or the inflammatory biomarker CRP [39–41]. The clinical variables included into the classical risk factor model can not be treated, but are indicators for an adverse outcome and mark patients with a higher risk for a secondary CV event [28, 29, 37]. However, strategies to manage patients with such a high risk are not known until now. Indeed, the results from our study suggest that guideline appropriate treatment should be strictly adhered to in these patients and there may be the need for even more aggressive medical treatment and close follow-up monitoring in those patients [16, 25, 27].

### Combining traditional risk factors and clinical variables to improve risk prediction

The salient finding of our study is that after combining the four clinical variables to the traditional risk factors, the C-index of the overall model for prediction of the outcome of the study was improved to 0.66 in the overall cohort, which was not achieved by each approach alone. Until now, there are only few guidelines describing the excess risk attributed to the discussed clinical variables but in our cohort all proved to be useful in predicting an adverse outcome [42].

### Limitations

There are limitations to the Athero*Gene* cohort which should be noted when interpreting the results. Every patient was examined only once, there were no follow-up visits at either department to follow medication use, blood pressure or treatment of diabetes. Patient follow-up was achieved by study personal analysing questionnaires about end-points during the study period. Baseline characteristics are available from initial presentation only.

### Conclusion

To conclude, traditional risk factors have a high impact to identify patients at risk for a secondary event in a cohort with already proven coronary artery disease. Nevertheless, occurrence of cardiovascular death and non-myocardial infarction were also influenced by clinical variables like ejection fraction and creatinine clearance as measure of renal function. Combining risk factors and clinical variables predicted outcome better than risk factors alone. Thus, this combined approach is superior.
